# Hemoglobin A1c and Weight Changes Associated With Grocery Delivery and Low-Carbohydrate Education in Adults With Type 2 Diabetes and Food Insecurity: Observational Quality Improvement Cohort Study

**DOI:** 10.2196/89459

**Published:** 2026-09-21

**Authors:** Marika Waselewski, Eric Waselewski, Lauren Oshman, Kaitlyn Hatch, Tammy Chang

**Affiliations:** 1 Department of Family Medicine, University of Michigan, 2800 Plymouth Rd Building 14-G128, Ann Arbor, MI, 48109, United States, 1 7349987124; 2 Department of Internal Medicine, University of Michigan, Ann Arbor, MI, United States; 3 Department of Family Medicine, Institute for Healthcare Policy and Innovation, University of Michigan, Ann Arbor, MI, United States; 4 Department of Clinical Quality, Michigan Medicine, Ann Arbor, MI, United States

**Keywords:** type 2 diabetes, hemoglobin A_1c_, HbA_1c_, food insecurity, food is medicine, grocery delivery, low-carbohydrate diet, education

## Abstract

**Background:**

Type 2 diabetes mellitus (T2D) is one of the most significant chronic health challenges in Michigan, especially for individuals with food insecurity. Improving nutrition, including lowering carbohydrate intake, is foundational to T2D prevention and management. Grocery delivery paired with low-carbohydrate education has the potential to reduce logistical barriers to dietary change but is not well understood in individuals with food insecurity.

**Objective:**

The objective of our study was to evaluate changes in clinical metrics of hemoglobin A_1c_ (HbA_1c_), weight, and diabetes medication use after a paired grocery delivery and low-carbohydrate education program.

**Methods:**

The Healthy Eating Jumpstart Program (hereafter, Jumpstart) enrolled adult Michiganders with T2D and low income or food insecurity from 21 Michigan primary care practices between October 2022 and May 2023. Jumpstart was a 3-month, nonrandomized grocery delivery and low-carbohydrate education quality improvement program without a control group. Participants received US $80 every month through Shipt to purchase eligible healthy foods, along with low-carbohydrate educational materials. Clinical metrics of HbA_1c_, weight, and diabetes medication use were collected from medical records from 6 months before enrollment through 12 months after enrollment. Changes in HbA_1c_ and weight from baseline were evaluated at 3, 6, 9, and 12 months, with stratification by baseline glycemic control and glucagon-like peptide-1 receptor agonist (GLP-1 RA) medication use, respectively. Overall change was assessed with paired *t* tests comparing baseline values to each participant’s latest value for HbA_1c_ and weight. Medication changes were assessed by comparing baseline and 12-month dosing to categorize changes as increased, decreased, eliminated, or unchanged.

**Results:**

A total of 83 patients participated in Jumpstart. Participants were primarily female (57/79, 72%), White (70/79, 89%), and had a high school degree or less (35/79, 44%). At baseline, the average HbA_1c_ was 7.6% (SD 2%), average weight was 105 (SD 25.8) kg, and average BMI was 38.2 (SD 8.8) kg/m^2^. The overall reduction in HbA_1c_ from baseline was 0.4% (SD 1.4%; *P*=.02; n=68), and this reduction was greater when limited to individuals with uncontrolled HbA_1c_ at baseline (mean 0.7%, SD 1.8; *P*=.02; n=38). The overall percent reduction in weight from baseline was 1.7% (SD 4.4%; *P*=.002; n=66), but this change was not significant when limited to non–GLP-1 RA users. Most medications had no change in dosing (77/166, 46%), and slightly more medications were increased or added (n=52, 31%) than decreased or discontinued (n=37, 22%).

**Conclusions:**

The results from this clinical evaluation suggest that the Jumpstart quality improvement program was associated with improvements in HbA_1c_ that persisted beyond 6 months and small reductions in weight among participants. A low-burden program such as Jumpstart may be a feasible approach to support long-term dietary change in patients with T2D and food insecurity or low income.

## Introduction

Type 2 diabetes mellitus (T2D) remains one of the most significant chronic health challenges in the United States, with more than 26 million (8%) US adults having a T2D diagnosis [1]. In Michigan, rates of T2D are slightly higher, with 12% of adults having a diabetes diagnosis and an additional 35% estimated to have prediabetes [2]. T2D disproportionately impacts individuals with low income, who experience both a high prevalence of the disease and increased rates of diabetes-related morbidity, mortality, and health care costs. In particular, food insecurity and low income are closely linked to suboptimal diet quality, an elevated risk of developing T2D, poorer diabetes management and glycemic control, increased rates of complications, and higher health care use [3].

Improving nutrition is foundational to T2D prevention and management. Low-carbohydrate dietary patterns (LCDs) are specifically noted by the American Diabetes Association (ADA) Consensus Report on Nutrition Therapy for their effects in improving glycemic control, promoting weight loss, and optimizing lipid profiles [4]. Healthier diets may also reduce reliance on antihyperglycemic medications and, in some cases, support diabetes remission [4-6]. A variety of programs aim to improve food security and diet quality, including food-is-medicine programs (medically tailored meals, food pantries, and produce prescriptions), diabetes self-management education, and participation in the federally funded Supplemental Nutrition Assistance Program (SNAP) and Special Supplemental Nutrition Program for Women, Infants, and Children (WIC) [3]. However, such programs often do not address the logistical challenges of obtaining healthy food or the gaps in nutritional knowledge faced by individuals with T2D and low income.

The rise of online grocery purchasing and delivery services presents an opportunity to address these logistical barriers [7,8]. Grocery delivery services have the potential to save time, promote planned purchasing, and eliminate transportation barriers; however, these services are often not used by individuals with low income [9]. In the growing food-is-medicine landscape [10-13], implementing and evaluating the clinical impact of programs with pragmatic designs is important for supporting sustainable and scalable solutions. Healthy Eating Jumpstart is a food-is-medicine program designed to jumpstart dietary change through short-term access to healthy food via grocery delivery paired with patient-directed diabetes education. We aimed to evaluate changes in clinical metrics of glycemic control, weight, and diabetes medication use after participation in the Healthy Eating Jumpstart quality improvement program among Michiganders with T2D and low income or food insecurity. We hypothesized that improving healthy food access through grocery delivery, combined with practical low-carbohydrate education, could reduce barriers to making dietary changes and support glycemic control and weight outcomes among adults with T2D experiencing food insecurity.

## Methods

### Program Overview

The Healthy Eating Jumpstart Program (hereafter, Jumpstart) was developed as a quality improvement initiative to address food access barriers among individuals with T2D and low income or food insecurity, recognizing that limited access to affordable, healthy foods may hinder implementation of recommended dietary changes. The program enrolled individuals with T2D and low income or food insecurity in a 3-month, nonrandomized grocery delivery and educational quality improvement program without a control group. Enrollment occurred between October 2022 and May 2023. The sample size was determined based on participant flow, budgetary constraints, and guidelines for feasibility pilot studies. Details of the program protocol are published elsewhere [14,15].

As a quality improvement evaluation, the program was assessed using a pragmatic pre-post design to evaluate changes in clinical outcomes. The evaluation is focused on within-participant changes rather than estimating causal effects. This manuscript follows the SQUIRE 2.0 (Standards for Quality Improvement Reporting Excellence) checklist [16] and is limited to the clinical metrics noted in the original protocol; other program measures will be published separately.

### Ethical Considerations

This program was developed for quality improvement purposes and was designated as not regulated by the University of Michigan Medical School Institutional Review Board (HUM00217985). After receiving an explanation of the program details, participants were asked to verbally confirm their interest in the program during enrollment and were prompted to sign a medical record release to allow access to their clinical data. Study IDs were assigned to participants to deidentify data for analysis. All data were collected and stored via secure university-approved iterations of Qualtrics (Qualtrics Inc) or REDCap (Vanderbilt University), phone calls, and printed surveys and were stored in the protected health information–compliant Michigan Medicine SharePoint [14]. Participants did not receive compensation for participating in the program beyond food credits (US $240 over 3 months) and a free 1-year Shipt membership.

### Program Setting

Participants were recruited from 21 primary care practices engaged in the Michigan Collaborative for Type 2 Diabetes (MCT2D), a statewide collaborative quality initiative focused on improving care for T2D [15]. The existing infrastructure of primary care teams, including case managers, social workers, and other team members at each participating practice, identified and recruited patients meeting inclusion criteria. The physician or advanced practice provider responsible for the patient’s care attested that the patient was safe to enroll in a low-carbohydrate dietary program.

### Participants

Individuals were eligible to enroll if they met the following criteria: age ≥18 years, a diagnosis of T2D, residence within a Shipt delivery zone, and at least one of the following indicators of low income—Medicaid insurance status, a positive screen for food insecurity, or self-reported household income <150% of the federal poverty level, based on household income and the number of people in the household. Diagnosis of type 1 diabetes, active pregnancy or breastfeeding, or use of a sodium-glucose cotransporter-2 inhibitor were reasons for exclusion.

### Intervention

Grocery delivery was selected for this intervention because transportation limitations, competing priorities, and affordability barriers may restrict access to healthy foods among individuals experiencing food insecurity. Participants received a 3-month Healthy Choice Allowance (US $80 per month) through the grocery delivery service Shipt to purchase healthy foods and have them delivered to their homes. Healthy Choice Allowance–eligible foods included fruits, vegetables, proteins, canned goods, and dairy and excluded items such as ice cream, pop, and candy. Participants also received low-carbohydrate educational materials during the program, including a comprehensive packet on nutrition skills, the basics of low-carbohydrate meal planning, and grocery shopping recommendations, as well as weekly e-newsletters highlighting recipes, nutrition information, and seasonal tips. All materials are publicly available online [14].

### Data Collection

**Figure 1.**
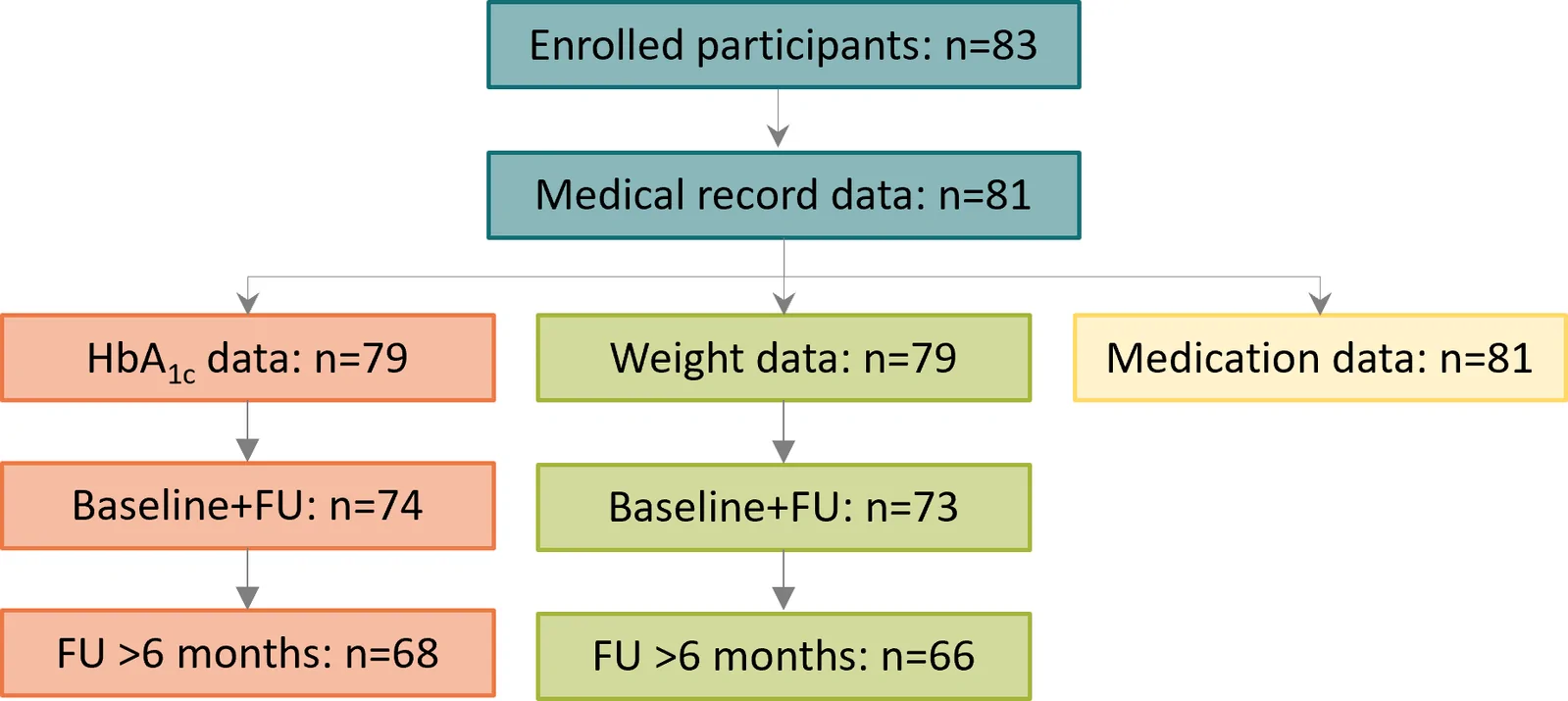
Flow diagram of participant medical record data availability. FU: follow-up; HbA_1c_: hemoglobin A_1c_.

Participants’ clinical data were abstracted from medical records at the primary care practice from 6 months prior to enrollment through 12 months after enrollment. Hemoglobin A_1c_ (HbA_1c_), weight, and glucose-lowering medications were reviewed and abstracted into a REDCap database for analysis. Because this program was implemented as a quality improvement initiative within routine primary care, participants were not expected to collect additional HbA_1c_ or weight measurements beyond routine care. Therefore, the data available and timing of measurements varied between individuals. Across all metrics, participants were excluded from the analysis if a baseline measurement was not available Figure 1. All participants were eligible for data collection, and none requested to be unenrolled from the program during the evaluation period.

### Data Analysis

Data management and preparation, including summary statistics, were conducted using Microsoft Excel for Microsoft 365 MSO (version 2606; Microsoft Corp). Statistical analyses were performed using SAS (version 9.4; SAS Institute Inc).

Analyses were designed to evaluate changes associated with participation in Jumpstart. As a nonrandomized quality improvement initiative without a comparison group, analyses focused on within-participant changes between baseline and follow-up. Findings are interpreted as associations rather than causal effects solely attributable to the intervention.

### Glycemic Control

Participant glycemic control was evaluated using HbA_1c_ measurements. HbA_1c_ data were only included in the analysis if the participant had a baseline HbA_1c_ as well as 1 additional measurement after enrollment (N=74). Participants were categorized as having controlled diabetes at baseline if their HbA_1c_ was <7% and uncontrolled diabetes if their HbA_1c_ was ≥7%. Glycemic control cutoffs were updated from 8% in the original protocol design to 7% to align with clinical metrics recommended by the ADA [17].

To evaluate changes in HbA_1c_, individual participant data were assigned to time points based on their enrollment date. Baseline HbA_1c_ was the closest data point to their enrollment date within 21 days after enrollment. Values for 3, 6, 9, and 12 months were selected as the latest laboratory value within each time frame. Longitudinal change in HbA_1c_ was calculated as the absolute percentage-point difference between each time point and baseline at 3, 6, 9, and 12 months. Change in HbA_1c_ was averaged for each time point across the cohort. These results were then stratified by baseline HbA_1c_ control status as an exploratory analysis because participants with HbA_1c_ ≥7% have greater potential for glycemic improvement.

An overall change in HbA_1c_ was also assessed. Each participant’s change in HbA_1c_ was calculated using their latest HbA_1c_ value, at or after 6 months, minus their baseline value. This approach was selected to reflect the pragmatic nature of the program evaluation, in which follow-up measurements were obtained through routine clinical care rather than at prespecified research visits. Statistical significance was then assessed using a paired *t* test (α=.05, 2-tailed). This test was repeated with stratification by baseline HbA_1c_ control status. A negative value for change in HbA_1c_ indicates a reduction in HbA_1c_. Normality of the difference scores was assessed using descriptive indices. Although data demonstrated some deviation from normality (skewness=−1.6; kurtosis=4.1), paired *t* tests were used due to their general robustness to moderate departures from normality given our sample size (N=68). Nonparametric methods (Wilcoxon signed-rank tests) were additionally conducted as sensitivity analyses.

### Weight and BMI

Participant weight and BMI were evaluated using height and weight measurements. Weight data were only included in the analysis if the participant had a baseline weight as well as 1 additional measurement after enrollment (n=73).

To evaluate changes in weight, individual participant data were assigned to time points based on their enrollment date. Baseline weight was the closest data point to their enrollment date within 21 days after enrollment. Values for 3, 6, 9, and 12 months were selected as the latest value within each time frame. Longitudinal change in weight was calculated as the percent change at 3, 6, 9, and 12 months, using the time point value minus the baseline value, divided by the baseline value. Percent change in weight was averaged for each time point across the cohort. These results were then stratified by glucagon-like peptide-1 receptor agonist (GLP-1 RA) status as an exploratory analysis due to their highly effective use for weight management. Participants were considered to be on a GLP-1 RA if they had any GLP-1 RA use documented during the 12-month program follow-up.

An overall percent change in weight was also assessed. Each participant’s percent change in weight was calculated using their latest weight value, at or after 6 months, as the latest value minus their baseline value, divided by baseline value. As with HbA_1c_, follow-up measurements reflected routine clinical care and therefore were not collected at uniform research-defined time points. Statistical significance was then assessed using a paired *t* test (α=.05, 2-tailed). This test was repeated with stratification by GLP-1 RA use. The percentage difference scores were approximately normally distributed (skewness=–0.2; kurtosis=0.6) for the full dataset. Consequently, paired *t* tests were used for the primary analyses, with nonparametric methods (Wilcoxon signed-rank tests) conducted as sensitivity analyses.

### Medication Use

Medications were categorized as active or inactive at baseline and consolidated into their medication classes. For example, basal and bolus insulin were consolidated into a category of insulin therapy, with total daily units calculated. Medication dosing at 12 months was compared to baseline, and the changes were categorized as increased, decreased, eliminated, or unchanged [18]. The addition of a new medication was considered an increased dose. Changes within the GLP-1 RA class were assessed using dose equivalencies between medications [19]. Participants whose medical records included no specified medication use were assumed to be taking no glucose-lowering medications. The percentage of medications with increased, decreased, eliminated, or unchanged dose over the evaluation period was then assessed across the cohort and by medication category.

## Results

### Overview

We enrolled a total of 83 participants between October 2022 and May 2023, with the program concluding at the end of July 2023. Participants primarily identified as female, White, and having a high school degree or less. At baseline, the mean HbA_1c_ was 7.6% (SD 2%), and the average weight was 105 (SD 25.8) kg. Participants primarily had a BMI ≥30.0 kg/m^2^, with a mean BMI of 38.2 (SD 8.8) kg/m^2^. Demographic characteristics of participants are reported in Table 1. All participants successfully placed at least 1 grocery order during the program and received mailed educational materials at their home.

**Table 1.** Demographic and clinical characteristics of program participants (N=83)^a^.

Characteristics	Participants^b^
Age (years) (n=81), mean (SD)^a^	56.8 (13.5)
Gender identity (n=79), n (%)	
Female	57 (72)
Male	22 (28)
Race (n=79), n (%)	
Black	4 (5)
Mixed race	1 (1)
Native American	1 (1)
Other	2 (3)
White	70 (89)
Hispanic (n=77), n (%)	
Yes	3 (4)
No	74 (96)
Education level (n=79), n (%)	
High school graduate or less	35 (44)
Some college or technical school	25 (32)
Associate’s or technical degree	8 (10)
Bachelor’s degree or higher	11 (14)
Food insecurity, n (%)	
Yes	67 (81)
No	16 (19)
Baseline HbA_1c_ (%; n=79), mean (SD)	7.6 (2)
Baseline HbA_1c_ control (n=79), n (%)	
Yes (<7%)	37 (47)
No (≥7%)	42 (53)
Baseline weight (kg; n=79), mean (SD)	105 (25.8)
Baseline BMI (kg/m^2^; n=79), mean (SD)	38.2 (8.8)
Baseline BMI category (n=79), n (%)	
Healthy weight (BMI 18.0-24.9 kg/m^2^)	3 (4)
Overweight (BMI 25.0-29.9 kg/m^2^)	15 (19)
Obese (BMI ≥30.0 kg/m^2^)	61 (77)
Baseline number of glucose-lowering medications (n=81), mean (SD)	1.8 (1.2)

a Not all data were available for all participants; where data were missing, appropriate sample size is specified.

b Values are presented as mean (SD) for continuous variables and n (%) for categorical variables.

Medical records were obtained for 81 (98%) of the 83 participants (Figure 1), including data from 1228 clinical encounters (excluding emergency medicine visits), 205 diabetes medications, 278 HbA_1c_ measurements, and 590 weight measurements. Encounter data included social work, care management, nutrition group classes, dietitian visits, diabetes education, podiatry, endocrinology, and primary care, among others.

### Glycemic Control

**Figure 2.**
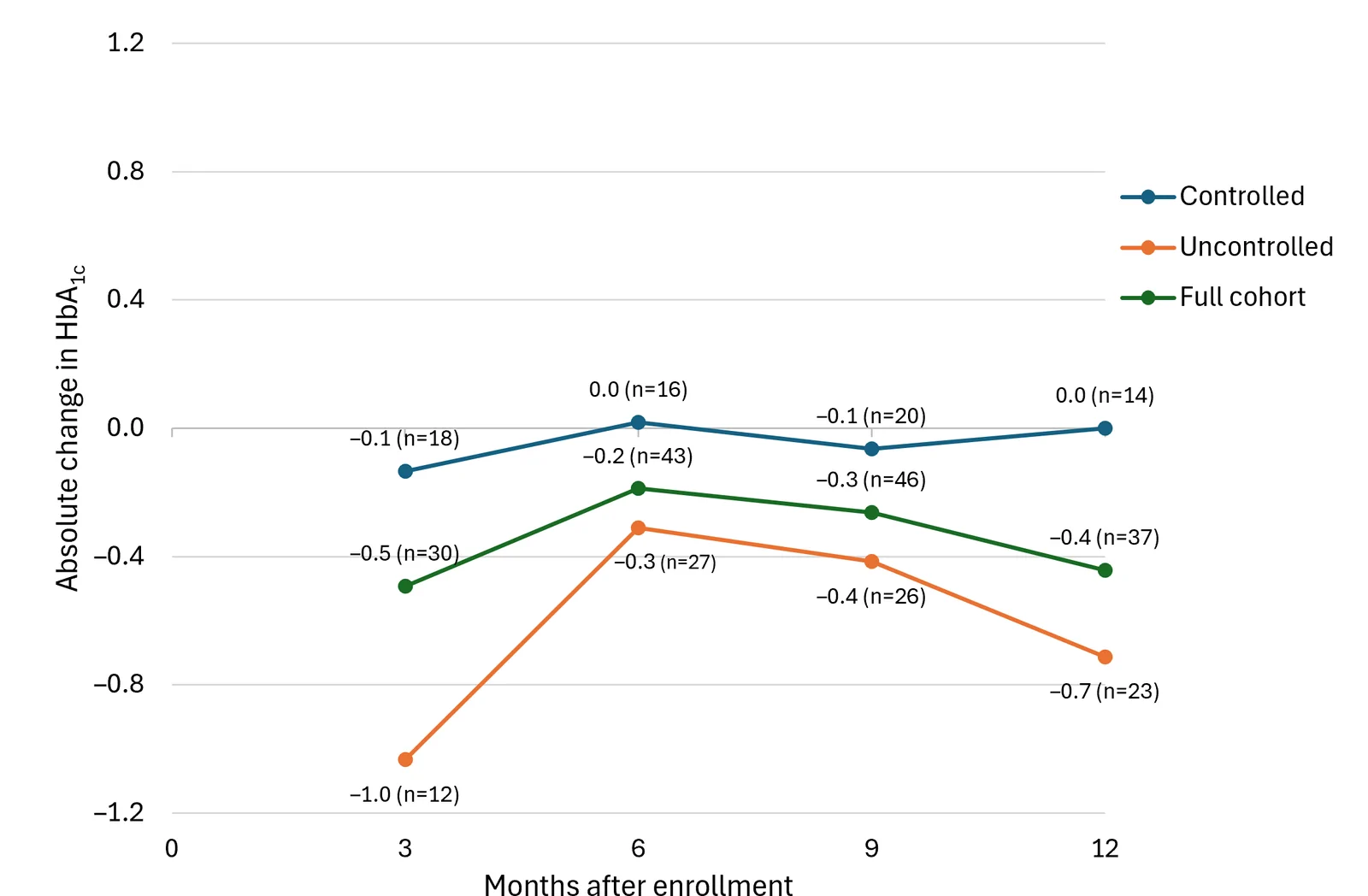
Change in hemoglobin A_1c_ (HbA_1c_) from baseline at 3, 6, 9, and 12 months for the full cohort and stratified by baseline glycemic control (HbA_1c_ <7%). Values are plotted as the absolute change from baseline, averaged across the cohort. N values at each time point indicate the number of participants with available data. No statistical comparisons were conducted.

HbA_1c_ follow-up data were available for 74 program participants; 55% (n=41) were categorized as having uncontrolled diabetes at baseline (HbA_1c_ ≥7%), and the mean HbA_1c_ was 7.7% (SD 2%) at baseline for this subset. At the end of follow-up, 54% (37/68) of individuals were categorized as having uncontrolled diabetes. Figure 2 (Multimedia Appendix 1) shows the average change in HbA_1c_ over time for the full cohort at each time point, as well as changes stratified by baseline HbA_1c_ control status.

The average overall reduction in HbA_1c_ from baseline was 0.4% (SD 1.4%; mean 7.7%, SD 2% to mean 7.3%, SD 1.6%; *t_67_*=–2.4; *P*=.02; *d*=–0.3; n=68). The mean interval from baseline to the latest HbA_1c_ measurement was 271 (SD 58, range 164-357) days. Individuals who were categorized as uncontrolled at baseline (n=38) had an average HbA_1c_ reduction of 0.7% (SD 1.8%; mean 8.9%, SD 1.9% to mean 8.2%, SD 1.5%; *t_37_*=–2.4; *P*=.02; *d*=–0.4). Individuals categorized as controlled at baseline (n=30) had a limited HbA_1c_ reduction of 0.1% (SD 0.8%; mean 6.2%, SD 0.5% to mean 6.1%, SD 0.9%; *t_29_*=–0.7; *P*=.51; *d*=–0.1). These results were similar in sensitivity analyses with nonparametric testing (signed-rank *P* values of .02, .04, and .20, respectively).

### Weight and BMI

**Figure 3.**
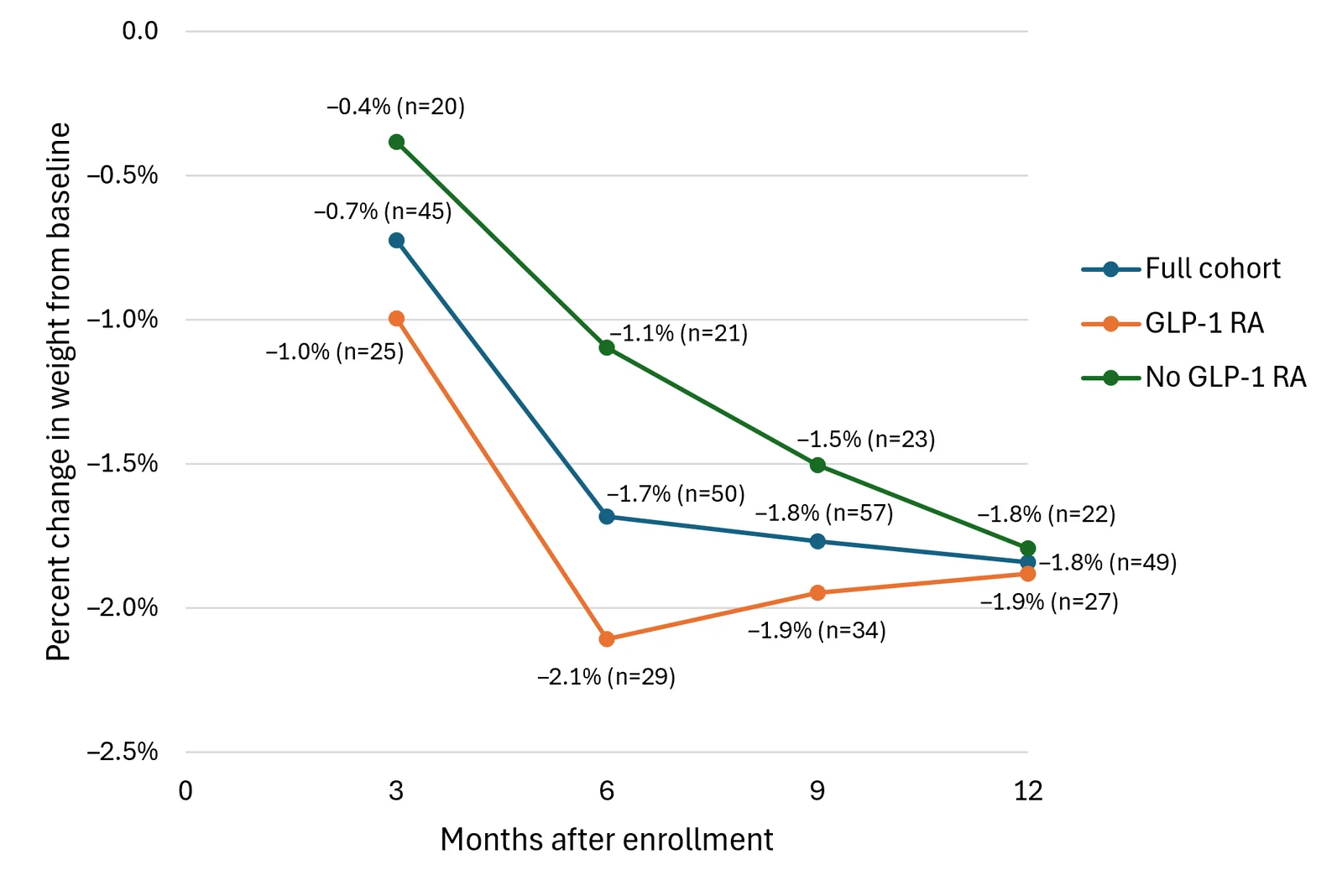
Percent change in weight from baseline at 3, 6, 9, and 12 months for the full cohort and stratified by glucagon-like peptide-1 receptor agonist (GLP-1 RA) use during the program. Values are plotted as the percent change from baseline, averaged across the cohort. N values at each time point indicate the number of participants with available data. No statistical comparisons were conducted.

Weight follow-up data were available for 73 program participants; 78% (n=57) had a BMI ≥30.0 kg/m^2^ (defined as obesity) at baseline, and 60% (n=44) were prescribed a GLP-1 RA medication at some point during the evaluation period. Figure 3 (Multimedia Appendix 2) shows the change in weight over time for the full cohort, as well as changes stratified by GLP-1 RA use.

The overall reduction in weight from baseline was 1.7 (SD 4.3, range –11.6 to 6) kg, which corresponded to a 1.7% (SD 4.4%) reduction (mean 105, SD 24.6 kg to mean 103.3, SD 24.8 kg; *t_65_*=–3.2; *P*=.002; *d*=–0.4; n=66). The mean interval from baseline to the latest weight measurement was 301 (SD 49, range 177-363) days. Individuals who had no GLP-1 RA use during the evaluation period had no detectable weight reduction during the evaluation, with a 1.1% (SD 4.4%) reduction (mean 99, SD 23.7 kg to mean 97.9, SD 23.5 kg; *t_28_*=–1.3; *P*=.19; *d*=–0.2; n=29), while those with GLP-1 RA use experienced statistically significant weight loss of 2.2% (SD 4.3%; mean 109.7, SD 24.6 kg to mean 107.5, SD 25.3 kg; *t_36_*=–2.2; *P*=.004; *d*=–0.5; n=37). These results were similar in sensitivity analyses with nonparametric testing (signed-rank *P* values of .005, .28, and .004, respectively).

### Medication Use

**Figure 4.**
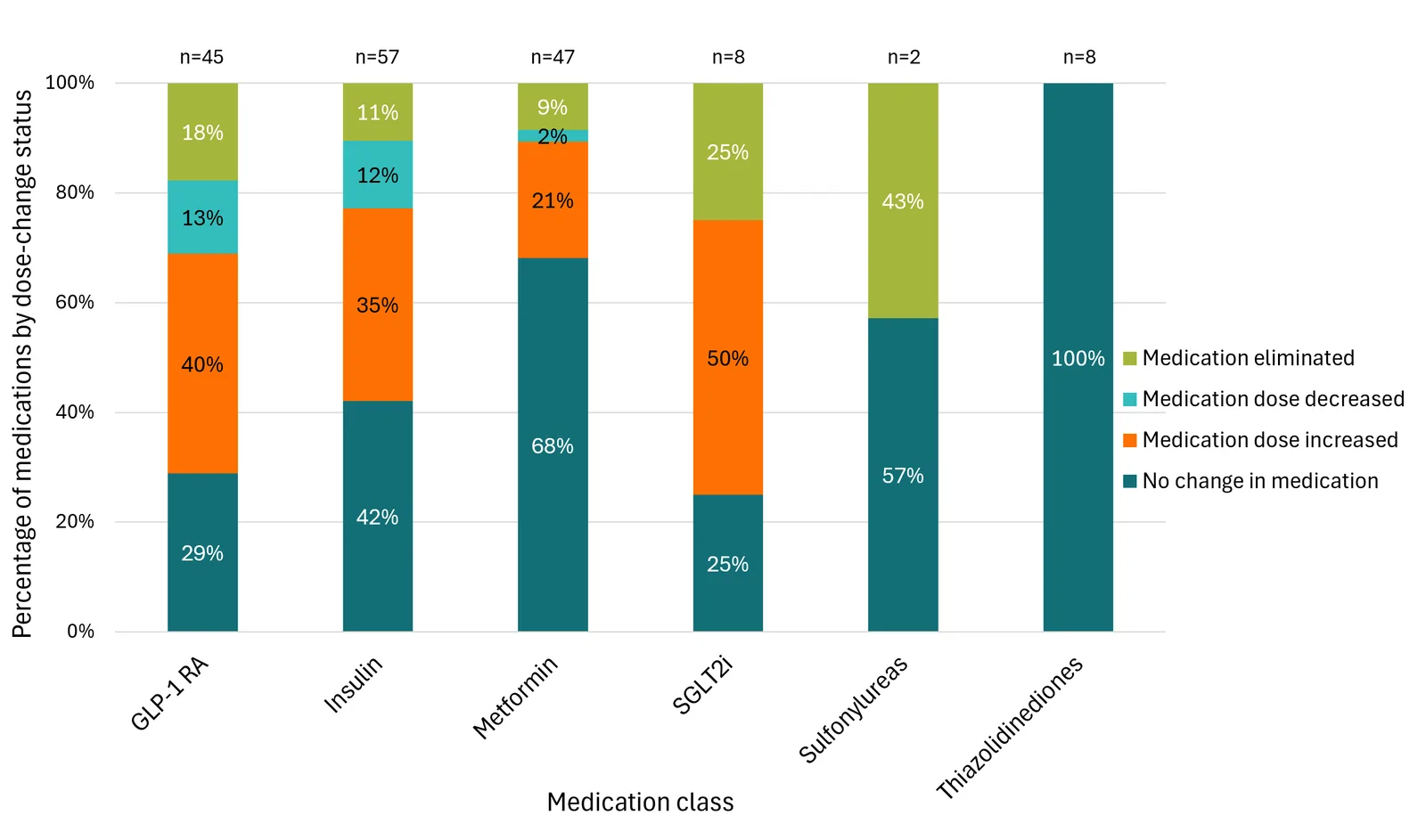
Medication use and dose modifications by medication class. Stacked bars represent the total number of users per class, with segments indicating dose changes from baseline to 12 months after enrollment. Totals are shown above each bar. No statistical comparisons were conducted. GLP-1 RA: glucagon-like peptide-1 receptor agonist; SGLT2i: sodium-glucose cotransporter 2 inhibitor.

At program enrollment, 72 (89%) of the 81 participants with medical record data were taking ≥1 glucose-lowering medication. At the end of the follow-up, 71 (88%) participants were taking glucose-lowering medications. Across the 74 participants who took 1 or more glucose-lowering medications during the program period, no change in dosing was most frequently observed (77/166, 46%), and slightly more medications were increased or added (n=52, 31%) than decreased or discontinued (n=37, 22%). Separating medication use by class, Figure 4 shows medication numbers and modifications during the evaluation period across these 74 patients (Multimedia Appendix 3).

## Discussion

### Principal Findings

Overall, several evaluated clinical metrics improved over the course of the program in line with our hypothesis. These changes were observed through up to 9 months of follow-up after the intervention period. The most significant clinical improvement was observed in glycemic control, with a small but statistically significant reduction in weight, and medications largely remained unchanged. These data suggest that the Jumpstart program supported participants in improving health outcomes through the provision of healthy foods and low-carbohydrate education.

The HbA_1c_ reduction of 0.4% at 6 to 12 months observed in our program is comparable to prior clinical trial data on LCDs, which reported reductions ranging from 0.09% to 0.61% at 3 to 6 months compared to controls [20,21]. However, prior meta-analyses indicate that these improvements are not sustained at 12 and 24 months [20,21], with HbA_1c_ values often returning toward baseline. In contrast, we did not observe evidence of an HbA_1c_ rebound after 6 months among participants with available data. The worsening of glycemic control in historical studies may partly reflect the highly restrictive clinical trial environment, resulting in low adherence to the intervention in the longer term [21-23]. The pragmatic design of the Jumpstart quality improvement program, emphasizing education to guide a shift toward a lower-carbohydrate dietary pattern rather than immediate adoption of a strict very low–carbohydrate diet, may have contributed to the absence of an observed HbA_1c_ rebound in our population. Participants with uncontrolled baseline HbA_1c_ had a larger observed reduction of 0.67%, suggesting that patients with higher baseline HbA_1c_ may experience greater improvement.

The change in weight after the Jumpstart program is less clear. Although a significant but small reduction in weight was noted across the full cohort, this result was attenuated when participants were stratified by GLP-1 RA use. This could indicate that GLP-1 RA use is a major contributor to weight loss rather than dietary changes. However, this stratification does not account for the duration of GLP-1 RA use, and individuals in the GLP-1 RA group could have been on the medication for more than 12 months (weight loss may have plateaued [24]) or may have taken only 2 doses during the evaluation period (which may not have been long enough to reach a therapeutic dose and influence weight [25]). Prior research has also noted suboptimal dietary intake among individuals taking GLP-1 RAs, suggesting that dietary interventions such as Jumpstart may complement pharmacotherapy and support healthier long-term eating patterns [26-28]. These findings do not rule out weight loss as a possible benefit of this program, but a more rigorous evaluation with a larger cohort would provide more information to isolate medication effects from dietary effects.

Beyond clinical impact, this program has several important strengths to note. The Healthy Eating Jumpstart provides insights into the impact and implementation of a pragmatic intervention in a high-risk population with low income and/or food insecurity that is often underrepresented in dietary intervention research. This program, administered through provider referrals at primary care clinics across the state of Michigan, suggests a feasible model for scaling combined food-is-medicine programs (food delivery+behavioral education) in the clinical setting. Successful implementation of food-is-medicine initiatives in the clinical setting is limited [12], despite the growing movement toward implementation. Pragmatic programs such as Jumpstart are needed. Health systems and community programs could consider evaluating similar dual-component interventions as a strategy to support clinical outcomes in highly vulnerable populations with diet-sensitive conditions. Additionally, this program was designed to be very low burden for patients, who simply receive access to food and educational content in their homes, without the need for additional clinic visits or counseling sessions.

### Limitations

The limitations of this program and its analysis are largely related to the observational design, limited sample size, and a lack of a control group in the cohort-based implementation. Because of these limitations, causal inference and generalizability are limited, and we were unable to more rigorously assess and control for medication changes or time-varying factors in our analyses. No adjustment was made for multiple testing, and findings with statistical significance should also be interpreted cautiously given the potential for type I error. The timing of the follow-up data points varied across participants because data were collected during routine clinical care. Consequently, the paired analyses are heterogeneous and reflect change from baseline to each participant’s latest available measurement rather than change at a standardized follow-up interval.

Clinic-specific variation in staffing, referral processes, and patient support may also influence program engagement and participant characteristics. Detailed food purchasing data and dietary intake were not captured in this manuscript, which limits a more direct connection between these factors and clinical metrics; however, all participants did use grocery credits during the program. No formal assessment of unintended consequences, such as participant burden or adverse dietary effects, was conducted. Additionally, although medical records included data from more than 1000 encounters, data from outside the primary care setting were limited, and records may have been incomplete, which could have resulted in missed medication changes, weight measurements, or laboratory values that would influence results. A larger-scale implementation with clinical evaluation beyond 12 months would help provide additional context on the sustainability of this program.

### Conclusions

The results from this clinical evaluation suggest that the Jumpstart quality improvement program was associated with improvements in HbA_1c_ beyond 6 months and small reductions in weight among participants with available follow-up data. Because this evaluation was observational and lacked a control group, causal effects of the program cannot be determined, and medication-related influences on weight and glycemic outcomes could not be fully isolated. A low-burden program such as Jumpstart may be a feasible approach to support long-term dietary change in patients with T2D and food insecurity or low income. Similar programs may also be beneficial in conjunction with medications for glycemic control and weight loss to support sustainable lifestyle changes and long-term health goals.

## Supplementary material

10.2196/89459 Multimedia Appendix 1 Average change (mean, SD) in hemoglobin A_1c_ from baseline to follow-up, stratified by baseline control status.

10.2196/89459 Multimedia Appendix 2 Average percent change (mean, SD) in weight from baseline to follow-up, stratified by any glucagon-like peptide-1 receptor agonist use during the evaluation period.

10.2196/89459 Multimedia Appendix 3 Medication classes and changes from baseline to 12 months of follow-up (n=74).

## Abbreviations

ADA: American Diabetes Association
GLP-1 RA: glucagon-like peptide-1 receptor agonist
HbA_1c_: hemoglobin A_1c_
LCD: low-carbohydrate diet
MCT2D: Michigan Collaborative for Type 2 Diabetes
SNAP: Supplemental Nutrition Assistance Program
SQUIRE 2.0: Standards for Quality Improvement Reporting Excellence
T2D: type 2 diabetes mellitus
WIC: Special Supplemental Nutrition Program for Women, Infants, and Children
