# Supplementary material for: Hemoglobin A1c and Weight Changes Associated With Grocery Delivery and Low-Carbohydrate Education in Adults With Type 2 Diabetes and Food Insecurity: Observational Quality Improvement Cohort Study
Source: JMIR Form Res. 2026 Sep 21;10:e89459. doi: 10.2196/89459 (PMC13592385; doi:10.2196/89459)
Supplement: Supplementary file 1 [file formative-v10-e89459-s001.docx]

**Multimedia Appendix 1: Average change in HbA1c from baseline to follow-up stratified by baseline control status, mean (standard deviation)**

| **Follow up months** | **All (n=74)** | **HbA1C <7.0% (controlled) at baseline (n=33)** | **HbA1C ≥7.0% (uncontrolled) at baseline (n=41)** |
| --- | --- | --- | --- |
| 0-3 months (n=30) | -0.5 (1) | -0.1 (0.6) | -1 (1.3) |
| 4-6 months (n=43) | -0.2 (1.1) | 0 (0.6) | -0.3 (1.3) |
| 7-9 months (n=46) | -0.3 (1.4) | -0.1 (1) | -0.4 (1.6) |
| 10-12 months (n=37) | -0.4 (1.6) | 0 (0.7) | -0.7 (2) |
|  | | | |
