# Supplementary material for: Hemoglobin A1c and Weight Changes Associated With Grocery Delivery and Low-Carbohydrate Education in Adults With Type 2 Diabetes and Food Insecurity: Observational Quality Improvement Cohort Study
Source: JMIR Form Res. 2026 Sep 21;10:e89459. doi: 10.2196/89459 (PMC13592385; doi:10.2196/89459)
Supplement: Supplementary file 2 [file formative-v10-e89459-s002.docx]

**Multimedia Appendix 2: Average percent change in weight from baseline to follow-up stratified by any GLP-1 RA use during the evaluation period, mean (standard deviation)**

| **Follow up months** | **All (n=73)** | **GLP-1 RA (n=44)** | **No GLP-1 RA (n=29)** |
| --- | --- | --- | --- |
| 0-3 months (n=45) | -0.7 (2.4) | -1.1 (3.4) | -0.1 (3.1) |
| 4-6 months (n=50) | -1.7 (3.3) | -2.1 (4.4) | -0.9 (3.3) |
| 7-9 months (n=57) | -1.8 (4.1) | -2.1 (4.1) | -1.2 (4.8) |
| 10-12 months (n=49) | -1.8 (4.3) | -2.1 (4.3) | -1.4 (4.5) |
