# Supplementary material for: Hemoglobin A1c and Weight Changes Associated With Grocery Delivery and Low-Carbohydrate Education in Adults With Type 2 Diabetes and Food Insecurity: Observational Quality Improvement Cohort Study
Source: JMIR Form Res. 2026 Sep 21;10:e89459. doi: 10.2196/89459 (PMC13592385; doi:10.2196/89459)
Supplement: Supplementary file 3 [file formative-v10-e89459-s003.docx]

**Multimedia Appendix 3: Medication classes and change from baseline to 12 months follow-up (n=74), n (%)**

| **Group** | **No Change** | **Dose increased** | **Dose decreased** | **Medication discontinued** |
| --- | --- | --- | --- | --- |
| GLP1-RA | 13 (28.9%) | 18 (40%) | 6 (13.3%) | 8 (17.8%) |
| Insulin | 24 (42.1%) | 20 (35.1%) | 7 (12.3%) | 6 (10.5%) |
| Metformin | 32 (68.1%) | 10 (21.3%) | 1 (2.1%) | 4 (8.5%) |
| SGLT2i | 2 (25%) | 4 (50%) | 0 (0%) | 2 (25%) |
| Sulfonylureas | 4 (57.1%) | 0 (0%) | 0 (0%) | 3 (42.9%) |
| Thiazolidinediones | 2 (100%) | 0 (0%) | 0 (0%) | 0 (0%) |
| **All medications** | 77 (46.4%) | 52 (31.3%) | 14 (8.4%) | 23 (13.9%) |
